# Association of a Palliative Surgical Approach to Stage IV Pancreatic Neuroendocrine Neoplasms with Survival: A Systematic Review and Meta-Analysis

**DOI:** 10.3390/cancers12082246

**Published:** 2020-08-11

**Authors:** Marina Tsoli, Maria-Eleni Spei, Göran Wallin, Gregory Kaltsas, Kosmas Daskalakis

**Affiliations:** 1 1st Department of Propaedeutic Internal Medicine, Endocrine Unit, National and Kapodistrian, University of Athens, 11527 Athens, Greece; martso.mt@gmail.com (M.T.); marilena_0108@hotmail.com (M.-E.S.); gkaltsas@endo.gr (G.K.); 2 Department of Surgery, Faculty of Medicine and Health, Örebro University, 701 85 Örebro, Sweden; goran.wallin@regionorebrolan.se

**Keywords:** neuroendocrine tumor, pancreas, palliative surgery

## Abstract

The role of primary tumor resection in patients with pancreatic neuroendocrine neoplasms (PanNENs) and unresectable distant metastases remains controversial. We aimed to evaluate the effect of palliative primary tumor resection (PPTR) on overall survival (OS) in this setting. We searched the MEDLINE, Embase, Cochrane Library, Web of Science and SCOPUS databases up to January 2020 and used the Newcastle–Ottawa scale (NOS) criteria to assess quality/risk of bias. A total of 5661 articles were screened. In 10 studies, 5551 unique patients with stage IV PanNEN and unresectable metastases were included. The five-year OS for PanNEN patients undergoing PPTR in stage IV was 56.6% vs. 23.9% in the non-surgically treated patients (random effects relative risk (RR): 1.70; 95% CI: 1.53–1.89). Adjusted analysis of pooled hazard ratios (HR) confirmed longer OS in PanNEN patients undergoing PPTR (random effects HR: 2.67; 95% CI: 2.24–3.18). Cumulative OS analysis confirmed an attenuated survival benefit over time. The complication rate of PPTR was as high as 27%. In conclusion, PPTR may exert a survival benefit in stage IV PanNEN. However, the included studies were subject to selection bias, and special consideration should be given to PPTR anchored to a multimodal treatment strategy. Further longitudinal studies are warranted, with long-term follow-up addressing the survival outcomes associated with surgery in stage IV disease.

## 1. Introduction

Pancreatic neuroendocrine neoplasms (PanNENs) are increasingly recognized and have a diverse clinical course, along with a variable metastatic propensity related to their biological behavior, the extent of disease and secretory status [1]. This is particularly important, as approximately 60% of patients with PanNENs present with distant metastases, mainly to the liver, commonly requiring a multidisciplinary treatment approach, including that of surgical resection [2]. However, in the majority of these patients, curative surgery is not feasible due to the pattern and extent of NEN metastases [3].

Patients with distant-stage disease may suffer from functioning tumors with severe associated clinical manifestations of hormonal excess, such as cases of metastatic gastrinoma, insulinoma, VIPoma, glucagonoma and somatostinoma, or non-functioning tumors that are more often detected at a late stage and may cause compressive symptoms to nearby structures or may even be incidentally detected [4].

With regards to surgery, in patients with locoregional disease only (stages I–III), resection of the primary PanNEN in non-functioning lesions > 20 mm and functioning lesions irrespective of the tumoral size is generally recommended when curative R0 resection is feasible. In the presence of familial syndromes with a genetic drive, such as in patients with multiple endocrine neoplasia type 1 (MEN1) or Von Hippel–Lindau syndrome (VHL), the surgical approach is modified accordingly, as per ENETS guidelines [5].

In stage IV disease, surgery may be considered with a curative intent in cases with resectable liver metastases and no extrahepatic extension, as well as with a palliative intent in selected cases where debulking of liver-dominant disease and/or non-surgical liver-targeted procedures may control tumor progression or ameliorate refractory secretory syndromes [5]. Although the currently available systemic and molecular targeted therapies for stage IV PanNENs have exhibited prime results with evident progression-free survival benefits [6], palliative resection of the primary tumor (PPTR) and the role of neoadjuvant systemic treatment in this setting remain controversial. Nevertheless, as well-differentiated PanNENs exhibit a relatively low growth rate and more favorable biological behavior compared to pancreatic adenocarcinoma, with five-year overall survival (OS) figures exceeding 60% in stage IV [2], more aggressive surgical strategies have been occasionally adopted, even in the presence of unresectable liver metastases [7].

There are limited data regarding the effect of PPTR in patients with unresectable stage IV PanNEN in respect of OS, surgical morbidity, symptomatic and biochemical improvement, as well as health-related quality of life preservation. The objective of the present systematic review and meta-analysis was to assess the outcomes of PPTR combined with standard multimodal treatment in patients with stage IV PanNENs, compared with standard multimodal treatment alone. The primary end point was overall survival (OS) and the secondary end point was the rate of complications following PPTR.

## 2. Results

### 2.1. Characteristics of Included Studies

**Figure 1:**
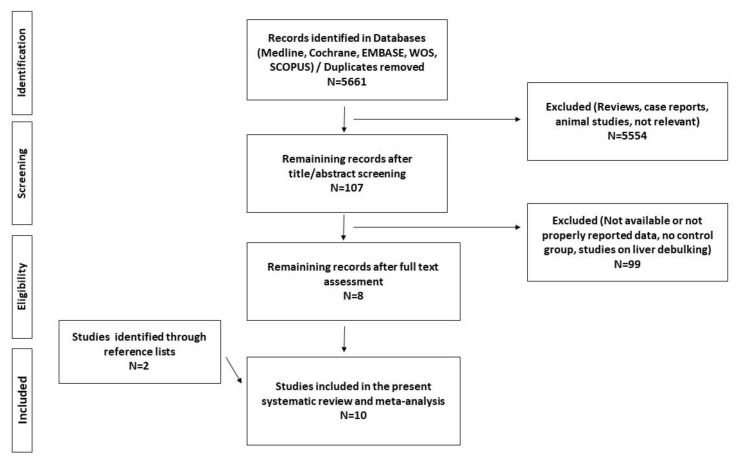
Preferred reporting items for systematic reviews and meta-analyses (PRISMA) flow diagram of the systematic review and quantitative meta-analysis.

We screened a total of 5661 articles. From 10 studies, 5551 unique patients with stage IV PanNEN and unresectable metastases were included. We followed the preferred reporting items for systematic reviews and meta-analyses (PRISMA) guidelines to conduct the literature search and the selection of included studies, as presented in the PRISMA flow diagram (Figure 1). The characteristics of the included studies are presented in Table 1, including information on potential conflicts of interest and funding [8,9,10,11,12,13,14,15,16,17].

### 2.2. Quality and Risk of Bias Assessment

The Newcastle–Ottawa scale (NOS) star template for quality assessment of each study is presented in Table 2. We could not identify any randomized controlled trials. All studies were retrospective observational cohort studies based on institutional or registry data. In two studies, a propensity score analysis had been applied.

To assess the presence of small study effects and the risk of reporting bias, effect size estimates from the included studies were plotted against the measure of each study’s size on funnel plots (Figures S1A and S2A (Supplementary Materials)). Potential reasons for funnel plot asymmetry could be the fact that a small number of studies were included (<10 studies in meta-analysis) and inter-study heterogeneity with respect to study quality, as depicted in NOS template (Table 2). Egger’s tests were conducted to thoroughly assess the distribution of the included studies in the funnel plots, and did not demonstrate any publication bias (Figures S1B and S2B (Supplementary Materials)).

### 2.3. Pooled Results for Unadjusted Five-Year Overall Survival (OS) Rates

**Figure 2:**
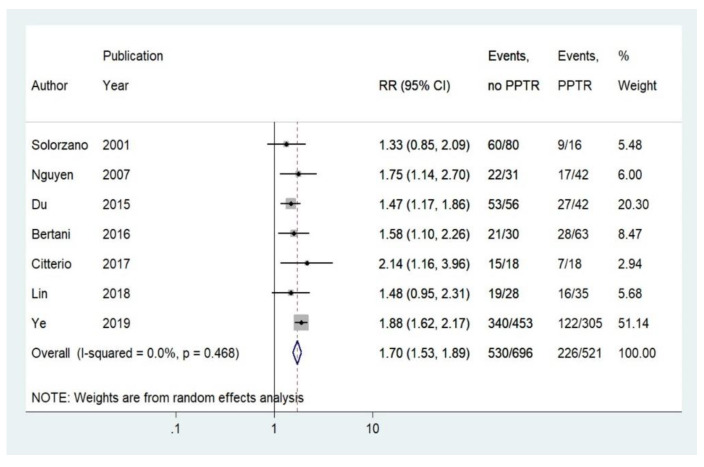
Forest plot comparing unadjusted 5-year overall mortality rates in stage IV PanNEN patients receiving non-surgical vs. palliative primary tumor resection (PPTR). Meta-analysis carried out using a random-effects model; relative risks are shown with 95% confidence intervals.

Seven studies reported five-year OS and were stratified by PPTR using relative risk (RR) analysis [8,11,12,14,15,16,17]. The five-year OS rate for patients undergoing PPTR was 56.6%, compared to 23.9% without PPTR (Figure 2; random effects relative risk (RR): 1.70; 95% CI: 1.53–1.89). There was no significant heterogeneity across the studies (I^2^ = 0%, *p*-value > 0.10). A funnel plot (Figure S1A (Supplementary Materials)) was also produced with no apparent evidence of asymmetry. Egger’s test (Figure S1B (Supplementary Materials)) and Begg’s test were performed, which gave no indication of publication bias (*p*-value = 0.35). We conducted a sensitivity analysis focusing on the impact of non-surgery in patients with PaNEN with overall mortality. In particular, the cumulative meta-analysis showed a relevant change between the estimates of RR by publication year in overall mortality (Figure S3 (Supplementary Materials)).

### 2.4. Pooled Results for Adjusted Overall Survival (OS) Rates

**Figure 3:**
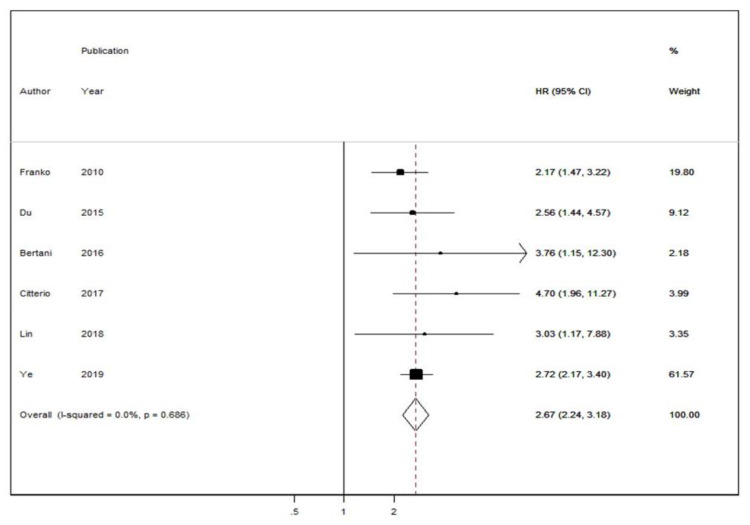
Forest plot comparing adjusted hazard ratios (HR) of overall mortality rate in stage IV PanNEN patients receiving non-surgical vs. palliative primary tumor resection (PPTR). Meta-analysis carried out using a random-effects model; Relative risks are shown with 95% confidence intervals.

**Figure 4:**
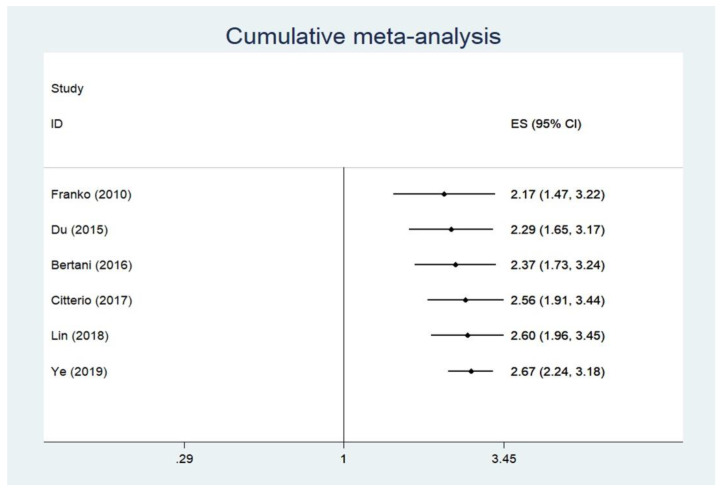
Cumulative adjusted survival meta-analysis by year of publication.

In six studies reporting Cox-regression multivariable survival analyses following PPTR or non-surgical treatment, a random-effects model hazard ratio (HR) of 2.67 (95% CIs: 2.24–3.18) was demonstrated in the PPTR group vs. the non-surgical group (Figure 3) [8,11,12,13,14,17]. There was no significant heterogeneity across the studies (I^2^ = 0%, *p*-value > 0.10). A funnel plot (Figure S2A (Supplementary Materials)) was also produced with no apparent evidence of asymmetry. Egger’s test (Figure S2B (Supplementary Materials)) and Begg’s test showed no indication of publication bias (*p*-value = 0.36). Finally, we conducted a sensitivity analysis focusing on the impact of non-surgery in patients with stage IV PanNENs with regards to overall mortality. This cumulative meta-analysis showed a relevant change between the estimates of HR by publication year in overall mortality (Figure 4).

### 2.5. Complication Rate of Palliative Primary Tumor Resection (PPTR)

The postoperative complication rate encountered in patients undergoing PPTR was reported in four studies (a total of 159 patients undergoing PPTR, inclusive of Whipple’s procedure). Overall postoperative morbidity was as high as 27% [8,9,14,15]. The complications’ severity ranged significantly, depending on the extent of surgery, patient performance status and other factors. However, its range was not appropriately reported, nor classified.

## 3. Discussion

Our systematic review and meta-analysis demonstrate that overall mortality rates at five years of follow-up of patients with stage IV PanNEN receiving palliative surgery and non-surgical treatment are as high as 43.4% and 76.1%, respectively. Unadjusted survival analysis revealed that patients not receiving PPTR had an almost 70% increased risk of death compared to the PPTR group. Adjusted HR multivariable survival analysis confirmed an approximately 2.7-fold increased risk of overall mortality in non-surgically treated patients compared to patients receiving PPTR. Thus, we were able to provide some evidence that PPTR may exert a survival benefit in stage IV PanNEN.

In addition, cumulative survival meta-analysis revealed an attenuated OS benefit of PPTR over time, possibly owing to recently available multimodal therapies for stage IV that are usually combined with surgery in the context of a multidisciplinary management of stage IV disease. This is indeed in accordance with the improved prognosis of PanNEN patients over time across all stages, which is evident in the latest Surveillance, Epidemiology and End Results (SEER) report [1]. Finally, the complication rate of palliative surgery in this meta-analysis was under-reported, as only four smaller-sample-size studies presented complication rates as high as 27%; however, they showed considerable variation in the extent of PPTR undertaken. Finally, our analysis could not assess outcomes, such as symptom-specific survival and health-related quality of life impairment following PPTR vs. non-surgical management due to insufficient studies addressing or properly reporting these issues.

All the included cohort studies achieved moderate to high quality (six studies scored ≥ seven stars) with respect to NOS criteria. However, all included studies suffered from selection bias, as depicted in the NOS template, including the ones applying a propensity score match methodology that failed to control for metastatic tumor burden and comorbidities. Inter-study heterogeneity was not observed in both the unadjusted and adjusted survival meta-analysis. Complementary testing did not reveal publication bias and small sample size effects in our meta-analysis. However, other confounders, such as selection bias and the precision of measurements, e.g., ICD (International Statistical Classification of Diseases and Related Health Problems)-coded data in SEER and National Cancer Database of the United States (NCBD) registry-based studies might have affected our findings [18]. In particular, the registry-based studies that were included in our meta-analysis lacked the granularity to identify certain subsets of patients who may derive the most benefit from surgery in the setting of stage IV disease. For example, data to determine proliferative markers including Ki-67, discrepancies in liver tumor burden and the presence of extrahepatic metastases, as well as detailed data on nonsurgical therapies, were not available [10,13,17]. Of note, although SEER- and NCBD-based studies might have considerable limitations regarding the strength of evidence, they still constitute a valuable source of information contributing to the present comprehensive summary of the available evidence [19].

Four institutional retrospective cohort studies that were included in our meta-analysis have addressed the extent of liver involvement. On multivariable Cox-regression OS analysis, high liver tumor load was confirmed as an independent prognostic factor in all four studies [8,11,12,16]. However, different criteria for liver tumor burden assessment were applied, such as cut-offs of 25% and 50%, as well as the sum of all metastatic loci in the liver being > 5 cm. Finally, although extrahepatic metastases have been linked with a worse prognosis, there were no evident differences in OS between patients with and without extrahepatic metastases in the two groups of our analysis in respect to OS [12]. In addition, six studies have provided data regarding pathology in the two groups we investigated and confirmed that increased Ki67 and poor differentiation were independent prognostic factors associated with worse OS in multivariable Cox-regression analysis [8,9,10,13,14,17]. Bertani et al. showed that a unit increment of the Ki67 index was significantly associated with a 7% greater risk of death [8]. Thus, well differentiated (WD)-PanNENs with lower tumor grades may benefit the most from PPTR. However, the likelihood of PPTR was highly dependent on tumor grade, accounting for a certain selection bias, as more patients with grade 1 and 2 tumors were resected compared to patients with higher tumor grades, hence introducing substantial collinearity into the Cox-regression models of the included studies.

Importantly, stage IV patients with extensive bilobar liver metastases and/or peritoneal carcinomatosis subjected to cytoreduction were not included in the scope of the present systematic review and meta-analysis. In addition, a recent systematic review and meta-analysis addressing palliative surgery in both small intestinal and pancreatic NENs in stage IV was published in 2017 [20]. Quality and risk of bias assessment, as well as unadjusted RR analysis, was not undertaken in that study. Nevertheless, since the publication of that study, a more recent SEER report with propensity score matched data became available [17], as well as three additional studies with a direct comparison between PPTR and non-surgical management in patients with stage IV PanNENs [10,11,14]. All these more recent studies confirmed that PPTR significantly prolonged OS in multivariable Cox-regression analysis, and these studies were included in our adjusted OS meta-analysis.

Although recommendations regarding PPTR in stage IV small intestinal NENs have recently been revisited, suggesting that removal of the primary tumor does not confer a survival benefit to asymptomatic patients [21], PPTR in stage IV PanNENs has never been fully recommended, except for selected low-risk patients with well-differentiated, low-proliferative tumors and locoregional compressive symptoms or severe intractable secretory manifestations [5]. Importantly, there is a higher rate of surgical morbidity for pancreatic compared to intestinal surgery, but also a less favorable prognosis of patients with stage IV PanNENs compared to that of patients with small intestinal NEN of the same stage [1].

With regards to primary tumor location in the head or the body-tail of the pancreas, resectability of the primary tumor represents the most commonly encountered selection bias for potentially eligible stage IV surgical candidates. For PanNENs located in the pancreatic head, resectability criteria are more narrow, as the tumor is often associated with invasion of the retroperitoneum, the celiac axis and/or vital structures in the hepatoduodenal ligament. Nevertheless, pancreatoduodenectomy for larger tumors in the the head of the pancreas is associated with higher postoperative morbidity and mortality, as compared to distal pancreatectomy. Therefore, distal pancreatectomies represent the majority of stage IV PanNEN cases subjected to PPTR in the included studies. In particular, Bertani et al. was the only study that excluded tumors located in the pacreatic head [8], whereas in the remaining nine studies all types of pancreatic resections were considered, with distal pancreatectomies making up the majority of procedures undertaken. Franco et al. hypothesized that enucleation is less effective in prolonging OS compared to formal pancreatic resections, despite being associated with better functional outcomes, but found no OS difference between the two operations [13]. Finally, in the latest SEER report by Ye et al., tumor location was not a prognostic factor for OS in propensity score-adjusted multivariable Cox-regression analysis [17].

In recent years, liver-targeted and systemic treatments, including peptide receptor radionuclide therapy, the mTOR inhibitor everolimus and the tyrosine kinase inhibitor sunitinib, have gained approval and have become more widely available. Thus, the scenario for the medical management of stage IV PanNENs may have changed during part of the time for the studies included in the present systematic review and meta-analysis. Importantly, the two groups that we compare in our meta-analysis appear to be heterogeneous with regards to nonsurgical therapies carried out. In particular, although the patients who underwent PPTR were more likely to receive liver-targeted therapies, and although there were also some discrepancies in systemic therapies, in 4 out of the 10 included studies chemo-embolization and/or systemic chemotherapy was specifically addressed in multivariable Cox-regression OS analysis and was not found to be an independent prognostic factor [8,9,14,16].

Our systematic review and meta-analysis had some limitations. Evolving classifications in the histopathology of PanNENs, as well as more sensitive modern imaging modalities, may have affected the selection of eligible patients for PPTR. In addition, the included studies are retrospective, and selection bias is very likely due to the assignment of patients for PPTR based on age, performance status, primary tumor resectability, grade and extent of metastatic disease. In two of the included studies, matched propensity-score patient data were used, and adjusted HR analysis was undertaken in six studies. However, none of the included studies took into consideration existing comorbidities that may have introduced selection bias into their analyses. Another limitation was the observed variation in the extent of PPTR performed in each of the included studies, along with the inclusion of both functioning and non-functioning PanNENs in some of the studies. Finally, although multiple medical therapies have become available for the management of stage IV PanNENs with evident effect in randomized controlled trials, head-to-head comparisons of such treatment modalities are not currently available and the multivariable analysis in most of the included studies could not include therapies commencing at different time points.

In the absence of high-quality prospective studies, we performed a systematic review and quantitative meta-analysis summarizing currently available evidence by applying a comprehensive search strategy, as well as a validated quality assessment protocol of the included contemporary literature to demonstrate a potential survival benefit of PPTR in patients with stage IV PanNENs.

## 4. Materials and Methods

### 4.1. Study Selection

Retrospective national registry-based and institutional cohort studies on patients with stage IV PanNENs undergoing surgery were assessed for eligibility. The following outcomes were required for eligibility: OS and postoperative complications following PPTR. Surgical series with a sample size of at least 10 PanNEN patients undergoing PPTR was necessary for study inclusion. Among multiple reports from the same national registry or institution with an overlap in patient cohorts of two studies, the latest eligible study was selected, unless these studies referred to different time periods. The PRISMA guidelines for reporting were followed [22].

### 4.2. Search Strategy

We conducted a systematic search in the Medline, Embase, Cochrane Library, Web of Science and SCOPUS databases for published and unpublished reports in any language to determine eligible studies. The full electronic search strategy and the search terms that we used are described in the supplemental data section (Table S1 (Supplementary Materials)). We examined full manuscripts of potentially eligible studies as necessary to finalize the study selection. Articles were independently evaluated by two of the authors (M.T. and K.D.) for relevance to the planned scope of the review. Reference lists of key publications were also reviewed for eligibility.

### 4.3. Data Extraction

Two of the authors (M.T. and K.D.) independently extracted the data used in this systematic review and meta-analysis. The primary outcome was defined as the overall mortality associated with PPTR in stage IV PanNENs and the secondary outcome was the complication rate of PPTR. We formulated the study hypothesis before data collection and resolved any discrepancies concerning the extracted data by consensus.

### 4.4. Risk of Bias

The classification of the included national registry and institutional studies followed the classical epidemiologic study design of cohort studies [19]. For quality assessment of the included studies we used the Newcastle-Ottawa scale (NOS) criteria [23]. The total score we applied using the NOS criteria ranged from 0 to 9 (worst to best). We assigned lower NOS scores to studies with a small sample size, ambiguity over PanNEN inclusion criteria, inadequate follow-up, lack of clarity over PPTR and more extensive debulking surgery, as well as failure to report multivariable Cox-regression survival analysis results and complication rates for patients undergoing surgery.

### 4.5. Statistical Analysis and Exploration of Heterogeneity

Statistical analyses were completed using the STATA statistical package (version 13.1; StataCorp, College Station, TX, USA). The pooled estimate for the association of PPTR in patients with stage IV PanNEN with the outcome of interest was evaluated by combining the study-specific relative risks (RRs) and hazard ratios (HRs) with random effects in the presence of heterogeneity. The random variance component was estimated using the approach by DerSimonian and Laird [24]. To explore heterogeneity between the studies, I^2^ statistics were used. When I^2^ was > 0.50% the statistical heterogeneity was considered substantial [25]. Publication bias and small study effects were assessed by visually inspecting funnel plots and conducting the Egger’s test to investigate potential asymmetry among the study estimates [18]. Furthermore, we performed sensitivity analysis by omitting one study at a time by publication year and assessing its effect on the overall summary of HRs as estimated before and after the exclusion of each study. A cumulative meta-analysis over year of publication was also performed [26].

## 5. Conclusions

The present study provides a systematic review and meta-analysis of a palliative surgical approach to patients with stage IV PanNENs, exhibiting an attenuated survival benefit of PPTR over time. Summarized contemporary evidence suggests that there may be a place for PPTR in selected patients with stage IV PanNENs who have low-volume liver tumor burden and lower grade tumors. In addition, as systemic and liver-targeted treatments have become more effective and more widely available, PPTR should be considered at the time of diagnosis, in combination with non-surgical therapies in the context of a personalized multimodal treatment strategy. Although survival seems to be positively affected by PPTR, our results should be interpreted with caution, due to the potential selection bias of the included studies. Complication rate following PPTR was relatively high; however, most recent and registry-based studies included in our systematic review did not report any surgery-related morbidity. Further well-designed longitudinal studies with longer follow-up, thoroughly controlling for confounders such as comorbidities, tumor grade and liver tumor burden are warranted. Such studies should also aim to evaluate the role of systemic treatments in a neoadjuvant setting or combined with resective surgery at any point, in order to delineate the role of surgery in stage IV PanNENs, both in terms of its survival impact and also in terms of elucidating aspects of treatment-related adverse effects and preservation of health-related quality of life. Until then, PPTR in patients with stage IV PanNENs and unresectable metastases should probably be considered in selected patients, anchored to a multidisciplinary treatment approach by a group of dedicated NEN specialists.

## Figures and Tables

**Table 1:** Characteristics of the included studies.

Adult Studies	Study Design	No of Patients (PanNEN, Stage IV, Submitted to PPTR)	Outcome(Patients with PanNEN)	Funding and Conflict of Interest
Bertani et al. [8]	Dual-center prospective cohort study (124 patients with PanNEN and liver metastases). Propensity score adjustment. Distal pancreatectomies only.	Intervention: 63 No intervention: 30	Median follow-up: Intervention: 96 mos, no intervention: 81 mos. Median OS: Intervention:111 mos; no intervention: 52 mos, HR of survival: 3.76 (1.15–12.3), *p* = 0.003. Liver tumor burden > 25% (HR: 5.03, *p* = 0.025) and Ki-67 (HR: 1.1, *p* < 0.001) affected survival. Overall complication rate: 16%.	No funding or conflict of interest reported.
Bettini et al. [9]	Single-center prospective cohort study (51 patients with metastatic non-functioning PanNEN). Both Whipple procedures and distal pancreatectomies.	Intervention:19 No intervention:32	Median follow-up: 26 mos. Median disease-related OS: Intervention: 54.3 mos; no intervention: 39.5 mos, *p* = 0.74. Median time to progression: Intervention: 7.6 mos, no intervention: 12 mos, *p* = 0.9. Poor differentiation (HR 3.01; 95% CI: 1.08–8.4, *p* = 0.035) and a Ki-67 ≥ 10% (HR: 4.4; 95% CI: 1.2–16.1, *p* = 0.023) associated with worse survival. Overall complication rate: 47%.	No conflict of interest reported. Support from the Associazione Italiana Ricerca Cancro (AIRC); European Community FP6 Program; Ministero Universit‘a e Ricerca e Ministero Salute, Rome, and Fondazione Giorgio Zanotto.
Chawla et al. [10]	NCDB-based retrospective cohort study (4038 patients with PanNEN)	Intervention: 167 No intervention: 3502	Median follow-up: Intervention: 14 mos, no intervention: 15.2 mos. Median survival time: Intervention: 71.8 mos, no intervention: 15.5 mos, *p* < 0.001 30-d mortality rate: Intervention: 7.6%, no intervention: 1.4% (*p* < 0.001). 90-d mortality rate: Intervention: 19.2%, no intervention: 4.3% (*p* < 0.001).	No funding or conflict of interest information mentioned in the article.
Citterio et al. [11]	Single-center retrospective cohort study (139 patients with liver metastases and functioning, well differentiated NEN). Functioning tumors only	Intervention: 18 No intervention: 18	Median OS: Intervention: 169 mos, no intervention: 18 mos, HR of survival: 4.7 (1.98–11.39), *p* < 0.0001. 5-year OS: Intervention: 61.1%, no intervention: 16.7%.	No funding or conflict of interest reported.
Du et al. [12]	Single-center retrospective cohort study (98 patients with PanNEN and liver metastases)	Intervention: 42 No intervention: 58	0.39 (0.22–0.70), *p* < 0.001. 5-year OS: Intervention: 35.7%, no intervention: 5.4%.	No funding or conflict of interest information mentioned in the article.
Franco et al. [13]	SEER-based cohort study (year 1973–2004; 2158 patients with non-functioning PanNEN)	Total: 614	Median OS: Intervention: 58 mos, no intervention: 12 mos, HR of death: 0.46 (0.31–0.68).	No funding or conflict of interest information mentioned in the article.
Lin et al. [14]	Single-center retrospective cohort study (129 patients with PaNEN and liver metastases)	Intervention: 35 No intervention: 28	Median follow-up: 37 mos. Median OS: Intervention: 72 mos, no intervention: 32 mos, HR of death: 0.33 (0.127–0.858), *p* = 0.01. Primary tumor resection the only significant prognostic factor for OS. Overall complication rate: 37%.	The authors reported no conflict of interest. Supported by the Chinese Academy of Medical Sciences Initiative for Innovative Medicine.
Nguyen et al. [15]	Single-center retrospective cohort study (73 patients with PanNEN). Both functional and non-functional tumors.	Intervention: 42 No intervention: 31	Median follow-up: 41 mos. 5-year OS: Intervention: 60%, no intervention: 30% (*p* = 0.025). Overall complication rate: 27%.	No funding or conflict of interest information mentioned in the article.
Solorzano et al. [16]	Single-center retrospective cohort study (163 patients with non-functioning PanNEN)	Intervention: 16 No intervention: 80	Median OS: Intervention: 36 mos, no intervention: 20 mos, *p* = 0.06. 5-year OS: Intervention: 56.3%, no intervention: 25%.	No funding or conflict of interest information mentioned in the article.
Ye et al. [17]	SEER-based cohort study (year 2004–2015; 1974 patients with stage IV non-functioning PanNEN) Propensity score adjustment.	Intervention: 305 No intervention: 60	Median follow-up: 19.5 mos. Median OS: Intervention: 79 mos, no intervention: 24 mos, HR = 0.368 (0.294–0.459), *p* < 0.0001. Median CSS; Intervention: 81 m (95% CI: 62.52–99.48), no intervention: 26 mos (95% CI: 21.22–30.78), *p* < 0.001. 5-year OS: Intervention: 60%, no intervention: 25%.	The authors reported no conflict of interest. Supported by the Natural Science Foundation of Ningbo, China, the Oncology Key Special Subject of Ningbo, and the Medical Scientific Research Foundation of Zhejiang Province.

Abbreviations: OS, Overall survival; CSS, Cancer-specific survival; HR, hazard ratio; NCDB, National Cancer Database of the United States; PanNEN, pancreatic neuroendocrine neoplasm; PPTR: Palliative resection of primary tumor; SEER, Surveillance, Epidemiology and End Results Program of the National Cancer Institute of the US.

**Table 2:** Newcastle–Ottawa scale (NOS) cohort star template.

Adult Studies	Selection	Comparability	Exposure
Bertani et al. [8]	***	**	**
Bettini et al. [9]	**	*	*
Chawla et al. [10]	***	*	*
Citterio et al. [11]	***	**	**
Du et al. [12]	***	**	**
Franco et al. [13]	***	**	**
Lin et al. [14]	***	**	***
Nguyen et al. [15]	***	*	**
Solorzano et al. [16]	***	*	**
Ye et al. [17]	***	**	**

The total Newcastle-Ottawa scale scores ranged from 0 (worst) to 9 (best) for the included studies, with a score of at least 6 indicating high quality.

## Supplementary Materials

The following are available online at https://www.mdpi.com/2072-6694/12/8/2246/s1, Table S1: Systematic literature search strategy; Figure S1A: Funnel plot for studies included in the comparison of stage IV PanNEN patients undergoing non-surgical treatment vs. patients receiving palliative resection of primary tumor (PPTR) with respect to unadjusted 5-year overall survival analysis, and S1B: Egger’s plot for studies included in this analysis; Figure S2A: Funnel plot for studies included in the comparison of stage IV PanNEN patients undergoing non-surgical treatment vs. patients receiving palliative resection of primary tumor (PPTR) with respect to adjusted hazard ratios (HRs) overall survival analysis, and S2B: Egger´s plot for studies included in this analysis; Figure S3: Cumulative unadjusted survival meta-analysis by year of publication with respect to 5-year overall survival analysis.
