# Supplementary material for: Association of a Palliative Surgical Approach to Stage IV Pancreatic Neuroendocrine Neoplasms with Survival: A Systematic Review and Meta-Analysis
Source: Cancers (Basel). 2020 Aug 11;12(8):2246. doi: 10.3390/cancers12082246 (PMC7464632; doi:10.3390/cancers12082246)
Supplement: Supplementary file 1 [file cancers-12-02246-s001.pdf]

Supplementary Materials: Association of a Palliative Surgical Approach to Stage IV Pancreatic Neuroendocrine Neoplasms with Survival: A Systematic Review and Meta-analysis

Marina Tsoli, Maria-Eleni Spei, Göran Wallin, Gregory Kaltsas and Kosmas Daskalakis

Table S1. Systematic Literature Search Strategy.

Medline.

	**Search Terms**	**Number of Studies**
**Pancreatic neuroendocrine tumour**		
1.	(pancreas or pancreatic or "islets of langerhans" or "Langerhans Islets").kf,tw.	242,942
2.	neuroendocrine.kf,tw.	49,506
3.	(tumo?r* or neoplasm* or cancer* or carcinom*).kf,tw.	3,024,241
4.	(neuroendocrine adj3 (tumo?r* or neoplasm* or cancer* or carcinom*)).tw,kf.	19,983
5.	1 and 4	5,804
6.	(pNET or PanNEN or PanNET or 'Non functioning pancreatic endocrine tumo?r*' or 'Non-functioning pancreatic endocrine tumo?r*' or 'Gastro-enteropancreatic neuroendocrine tumo?r*' or 'Gastroenteropancreatic neuroendocrine tumo?r*' or 'adenoma islet cell' or insulinoma or 'carcinom* islet cell' or gastrinoma or glucagonoma or somatostatinoma or vipoma or 'Zollinger Ellison Syndrome' or 'pancreas islet cell tumo?r*' or 'pancreas islet cell carcinom*').kf,tw.	12,681
7.	5 or 6	16,874
8.	pancreas/ or "islets of langerhans"/ or glucagon-secreting cells/ or insulin-secreting cells/ or pancreatic polypeptide-secreting cells/ or somatostatin-secreting cells/	112,446
9.	1 or 8	272,818
10.	exp Neuroendocrine Tumors/ or Multiple Endocrine Neoplasia Type 1/	166,962
11.	4 or 10	175,068
12.	'pancreatic incidentaloma'.kf,tw.	15
13.	pancreatic neoplasms/	70,785
14.	adenoma, islet cell/ or insulinoma/ or carcinoma, islet cell/ or gastrinoma/ or glucagonoma/ or somatostatinoma/ or vipoma/	9,949
15.	1 and 3	89,457
16.	12 or 13 or 15	107,898
17.	2 and 16	6,997
18.	7 or 14	21,651
19.	9 and 11	8,606
20.	17 or 18 or 19	24,284
Surgery		
21.	(Surgery or 'surgical resection' or pancreatectomy or pancreatectomies or enucleation or 'pancreatic resection' or 'pancreatic duodenectomy' or 'pancreaticoduodenectomy' or 'partial resection' or Pancreaticojejunostomy or 'surgical oncology').kf,tw.	1,237,589
22.	general surgery/ or surgical oncology/	38,600
23	Pancreatectomy/	12,928
24.	Pancreaticojejunostomy/	1,163
25.	Pancreaticoduodenectomy/	7,615
26.	21 or 22 or 23 or 24 or 25	1,265,975
Metastases		
27.	(metastases or metastasis or 'distant-stage disease' or 'stage 4' or 'stage IV').kf,tw.	359,836
28.	exp Neoplasm Metastasis/	198,240
29.	27 or 28	459,771
Combined sets		
30.	20 and 26 and 29	1,444
After deduplication		
31.	20 and 26 and 29	1,338

Cochrane.

	Search Terms	Number of Studies
**Pancreatic neuroendocrine tumour**		
#1	(pancreas or pancreatic or 'islets of langerhans' or 'Langerhans Islets'):ti,ab,kw	12,897
#2	(neuroendocrine):ti,ab,kw	2,559
#3	(tumo?r* or neoplasm* or cancer* or carcinom*):ti,ab,kw	201,195
#4	(neuroendocrine NEAR/2 (tumo?r* or neoplasm* or cancer* or carcinom*)):ti,ab,kw	1004
#5	#1 AND #4	477
#6	(pNET or PanNEN or PanNET or 'Non functioning pancreatic endocrine tumo?r*' or 'Non-functioning pancreatic endocrine tumo?r*' or 'Gastro-enteropancreatic neuroendocrine tumo?r*' or 'Gastroenteropancreatic neuroendocrine tumo?r*' or 'adenoma islet cell' or insulinoma or 'carcinoma islet cell' or gastrinoma or glucagonoma or somatostatinoma or vipoma or 'Zollinger Ellison Syndrome' or 'pancreas islet cell tumo?r' or 'pancreas islet cell carcinom*'):ti,ab,kw	876
#7	#5 OR #6	1,101
#8	MeSH descriptor: [Pancreas] this term only	435
#9	MeSH descriptor: [Islets of Langerhans] this term only	283
#10	MeSH descriptor: [Glucagon-Secreting Cells] this term only	10
#11	MeSH descriptor: [Insulin-Secreting Cells] this term only	371
#12	MeSH descriptor: [Pancreatic Polypeptide-Secreting Cells] explode all trees	1
#13	MeSH descriptor: [Somatostatin-Secreting Cells] this term only	0
#14	#8 OR #9 OR #10 OR #11 OR #12 OR #13	1,078
#15	#1 OR #14	13,153
#16	MeSH descriptor: [Neuroendocrine Tumors] explode all trees	2,037
#17	MeSH descriptor: [Multiple Endocrine Neoplasia Type 1] this term only	9
#18	#4 OR #16 OR #17	2,859
#19	(pancreatic incidentaloma):ti,ab,kw	0
#20	MeSH descriptor: [Pancreatic Neoplasms] this term only	1,526
#21	MeSH descriptor: [Adenoma, Islet Cell] this term only	20
#22	MeSH descriptor: [Insulinoma] this term only	11
#23	MeSH descriptor: [Carcinoma, Islet Cell] this term only	5
#24	MeSH descriptor: [Gastrinoma] this term only	6
#25	MeSH descriptor: [Glucagonoma] this term only	2
#26	MeSH descriptor: [Somatostatinoma] this term only	3
#27	MeSH descriptor: [Vipoma] this term only	2
#28	#21 OR #22 OR #23 OR #24 OR #25 OR #26 OR #27	34
#29	#1 AND #3	6,379
#30	#29 OR #20 or #19	6,379
#31	#2 AND #30	489
#32	#15 AND #18	490
#33	#7 OR #28	1,101
#34	#31 OR #32 OR #33	1,122
Metastases		
#35	(metastases or metastasis or 'distant-stage disease' or 'stage 4' or 'stage IV'):ti,ab,kw	56,916
#36	MeSH descriptor: [Neoplasm Metastasis] explode all trees	4,846
#37	#35 OR #36	57,057
Surgery		
#38	(Surgery or 'surgical resection' or pancreatectomy or pancreatectomies or enucleation or 'pancreatic resection' or 'pancreatic duodenectomy' or 'pancreaticoduodenectomy' or 'partial resection' or Pancreaticojejunostomy or 'Surgical Oncology'):ti,ab,kw	203,412
#39	MeSH descriptor: [General Surgery] explode all trees	347
#40	MeSH descriptor: [Surgical Oncology] explode all trees	2
#41	MeSH descriptor: [Pancreatectomy] explode all trees	168
#42	MeSH descriptor: [Pancreaticojejunostomy] explode all trees	61
#43	MeSH descriptor: [Pancreaticoduodenectomy] explode all trees	243
#44	#38 OR #39 OR #40 OR #41 OR #42 or #43	203,412
Combined sets		
#45	#34 AND #37 AND #44	82
After deduplication		
#46	#34 AND #37 AND #44	53

Embase.

	Search Terms	Number of Studies
**Pancreatic neuroendocrine tumour**		
1.	'gastroenteropancreatic neuroendocrine tumor'/de OR 'pancreas islet cell tumor'/exp OR ('pancreas islet cell tumo*r*':ti,ab,kw OR 'pancreatic neuroendocrine tumo*r*':ti,ab,kw OR 'pancreatic neuroendocrine neoplasm*':ti,ab,kw OR 'gastroenteropancreatic neuroendocrine tumo*r*':ti,ab,kw OR 'neuroendocrine tumo*r* of the pancreas':ti,ab,kw OR 'pnet':ti,ab,kw OR 'pannen':ti,ab,kw OR 'pannet':ti,ab,kw OR 'non-functioning pancreatic endocrine tumo*r*':ti,ab,kw OR 'adenoma islet cell*':ti,ab,kw OR insulinoma:ti,ab,kw OR 'carcinoma islet cell*':ti,ab,kw OR gastrinoma:ti,ab,kw OR glucagonoma:ti,ab,kw OR somatostinoma:ti,ab,kw OR vipoma:ti,ab,kw OR 'pancreas islet cell carcinom*':ti,ab,kw OR 'zollinger ellison syndrome':ti,ab,kw)	31,778
2.	pancreas:ti,ab,kw OR pancreatic:ti,ab,kw OR 'pancreas islets':ti,ab,kw OR 'islets of langerhans':ti,ab,kw OR 'langerhans islets':ti,ab,kw	337,247
3.	(neuroendocrine NEAR/3 carcinom*) OR (neuroendocrine NEAR/3 tumo*r*) OR (neuroendocrine NEAR/3 cancer*) OR (neuroendocrine NEAR/3 neoplasm*):ti,ab,kw	35,275
4.	2. AND 3.	11,673
5.	'endocrine pancreas'/exp OR 'pancreas islet'/exp OR ('pancreas islets':ti,ab,kw OR 'islets of langerhans':ti,ab,kw OR 'glucagon-secreting cells':ti,ab,kw OR 'insulin-secreting cells':ti,ab,kw OR 'pancreatic polypeptide-secreting cells':ti,ab,kw OR 'somatostatin-secreting cells':ti,ab,kw OR 'endocrine pancreas':ti,ab,kw)	74,012
6.	'neuroendocrine tumor'/de OR 'multiple endocrine neoplasia type 1'/de OR ('multiple endocrine neoplasia type 1'	24,768
7.	5. AND 6.	1,010
8.	'pancreas tumor'/de OR 'pancreas adenoma'/de OR ('pancreas cancer'/exp OR 'pancreatic incidentaloma'/de OR 'pancreas tumo*r*':ti,ab,kw OR 'pancreas adenoma':ti,ab,kw OR 'pancreas cancer':ti,ab,kw OR 'pancreatic incidentaloma':ti,ab,kw OR 'pancreas carcinom*'):ti,ab,kw	126,590
9.	neuroendocrine:ti,ab,kw	
10.	8 AND 9	4,715
11.	1. OR 4. OR 7. OR 10.	36,755
Metastases		
12.	'metastasis'/exp OR 'metastasis':ti,ab,kw OR 'metastases':ti,ab,kw OR 'distant-stage disease*':ti,ab,kw OR 'stage 4':ti,ab,kw OR 'stage iv':ti,ab,kw	781,010
Surgery		
13.	'surgery'/exp OR 'pancreas surgery'/de OR 'pancreatectomy'/exp OR 'surgical oncology'/de OR 'enucleation'/exp OR 'pancreaticoduodenectomy'/de OR 'pancreaticojejunostomy'/de OR 'partial resection'/exp OR (surgery:ti,ab,kw OR 'pancreas surgery':ti,ab,kw OR 'surgical resection':ti,ab,kw OR 'pancreatectomy':ti,ab,kw OR 'enucleation':ti,ab,kw OR 'pancreatic resection':ti,ab,kw OR 'pancreatic duodenectomy':ti,ab,kw OR 'pancreaticojejunostomy':ti,ab,kw OR 'partial resection':ti,ab,kw OR 'surgical oncology':ti,ab,kw	5,232,687
Combined sets		
14.	11. AND 12. AND 13.	4,925
After deduplication		
15.	11. AND 12. AND 13.	2,533

Web of Science.

	Search terms	Number of studies
**Pancreatic neuroendocrine tumour**		
1.	TS=(pancreas or pancreatic or "islets of langerhans" or "Langerhans Islets")	275,651
2.	TS=(neuroendocrine NEAR/3 carcinom*) OR (neuroendocrine NEAR/3 tumo*r*) OR (neuroendocrine NEAR/3 cancer*) OR (neuroendocrine NEAR/3 neoplasm*)	28,436
3.	1. AND 2.	8,117
4.	TS=(neuroendocrine)	60,719
5.	TS=(pNET or PanNEN or PanNET or "Non functioning pancreatic endocrine tumo*r*" or "adenoma islet cell*" or insulinoma or "carcinoma islet cell*" or gastrinoma or glucagonoma or somatostatinoma or vipoma or "pancreas islet cell tumo*r*" OR "pancreatic neuroendocrine tumo*r*" OR "pancreatic neuroendocrine neoplasm*" OR	16,913

	"pancreatic neuroendocrine cancer*" OR "gastroenteropancreatic neuroendocrine tumo*r*" OR "gastro-enteropancreatic neuroendocrine tumo*r*" OR "neuroendocrine tumo*r* of the pancreas" OR "pancreas islet cell carcinom*" OR "zollinger ellison syndrome")	
6.	TS=("glucagon-secreting cells" or "insulin-secreting cells" or "pancreatic polypeptide-secreting cells" or "somatostatin-secreting cells")	1,681
7.	1. OR 6.	276,164
8.	TS=("neuroendocrine tumo*r*" OR "multiple endocrine neoplasia type 1")	21,602
9.	7. AND 8.	7,497
10.	TS=("pancreas tumor" OR "pancreas adenoma" OR "pancreas cancer" OR "pancreatic incidentaloma" OR "pancreas carcinom*")	1,984
11.	4. AND 10.	47
12.	3. OR 5. OR 9. OR 11.	20,108
Metastases		
13.	TS=(metastases or metastasis or "distant-stage disease" or "stage 4" or "stage IV")	379,169
Surgery		
14.	TS=(surgery or "surgical resection" or pancreatectomy or pancreatectomies or enucleation or "pancreatic resection" or "pancreatic duoenectomy" or pancreaticoduodenectomy or pancreaticojejunostomy or "partial resection" or "surgical oncology")	1,151,299
Combined sets		
15.	12. AND 13. AND 14.	1,211
After deduplication		
16.	12. AND 13. AND 14.	233

Scopus.

	**Search Terms**	**Number of Studies**
**Pancreatic neuroendocrine tumour**		
1.	TITLE-ABS-KEY(neuroendocrine W/3 tumo*r*) OR (neuroendocrine W/3 cancer*) OR (neuroendocrine W/3 neoplasm* OR neuroendocrine W/3 carcinom*)) **AND** (TITLE-ABS-KEY(pancreas OR pancreatic OR "islets of langerhans" OR "Langerhans Islets")	11,100
2.	((TITLE-ABS-KEY("glucagon-secreting cells" OR "insulin-secreting cells" OR "pancreatic polypeptide-secreting cells" OR "somatostatin-secreting cells" OR pancreas OR pancreatic OR "islets of langerhans" OR "Langerhans Islets") **AND** TITLE-ABS-KEY("neuroendocrine tumo*r" OR "multiple endocrine neoplasia type 1")))	10,098
3.	(((TITLE-ABS-KEY("pancreas adenoma" OR "pancreas cancer" OR "pancreas tumo*r*" OR "pancreatic incidentaloma" OR "pancreas carcinom*") **AND** TITLE-ABS-KEY(neuroendocrine)))	1,742
4.	((TITLE-ABS-KEY(pnet OR pannen OR pannet OR "non functioning pancreatic endocrine tumo*r*" OR "adenoma islet cell*" OR insulinoma OR "carcinoma islet cell*") OR TITLE-ABS-KEY(gastrinoma OR glucagonoma OR somatostatinoma OR vipoma OR "pancreas islet cell tumo?r*" OR "gastroenteropancreatic neuroendocrine tumo?r*")OR TITLE-ABS-KEY("neuroendocrine tumo*r* of the pancreas" OR "pancreas islet cell carcinom*" OR "zollinger ellison syndrome")))	23,585
5.	1. OR 2. OR 3. OR 4.	31,856
Metastases		
6.	((TITLE-ABS-KEY (metastases OR metastasis OR "distant-stage disease" OR "stage 4" OR "stage IV"))	659,413
Surgery		
7.	(TITLE-ABS-KEY (surgery OR "surgical resection" OR pancreatectomy OR pancreatectomies OR enucleation OR "pancreatic resection" OR "pancreaticojejunostomy" OR "pancreaticoduodenectomy" OR "partial resection" OR "surgical oncology")	2,280,919
Combined sets		
8.	5. AND 6. AND 7.	2,716
After dedublication		
9.	5. AND 6. AND 7.	1,454

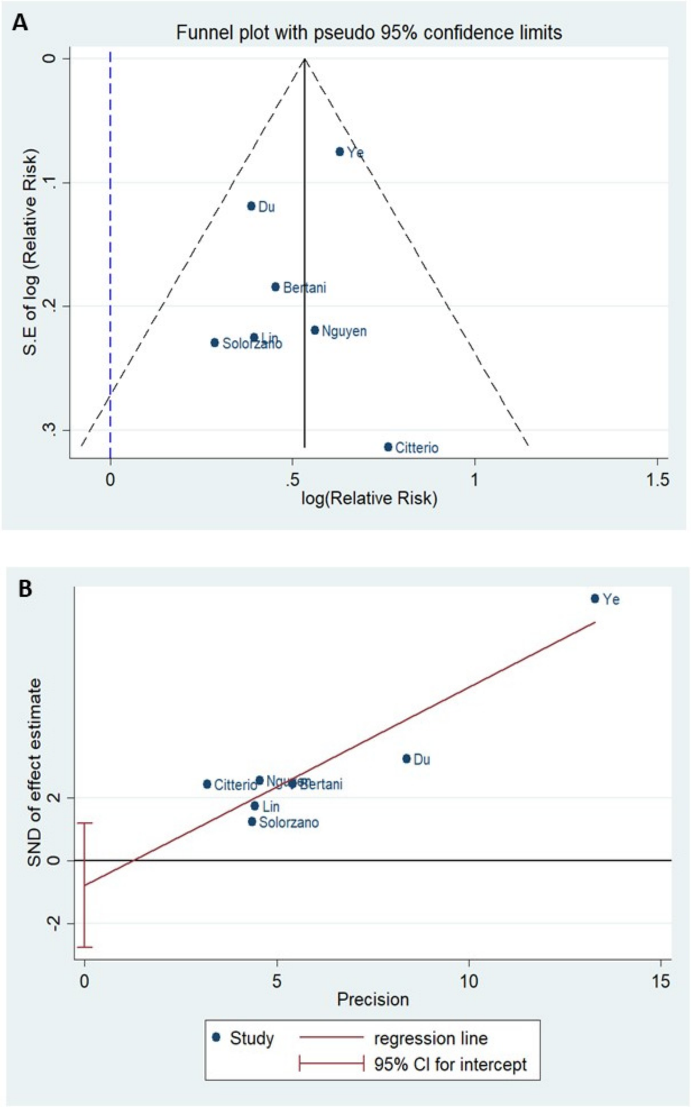

Figure S1. **A**: Funnel plot for studies included in the comparison of stage IV PanNEN patients undergoing non-surgical treatment vs. patients receiving palliative resection of primary tumor (PPTR) with respect to unadjusted 5-year overall survival analysis; and **B**: Egger´s plot for studies included in this analysis.

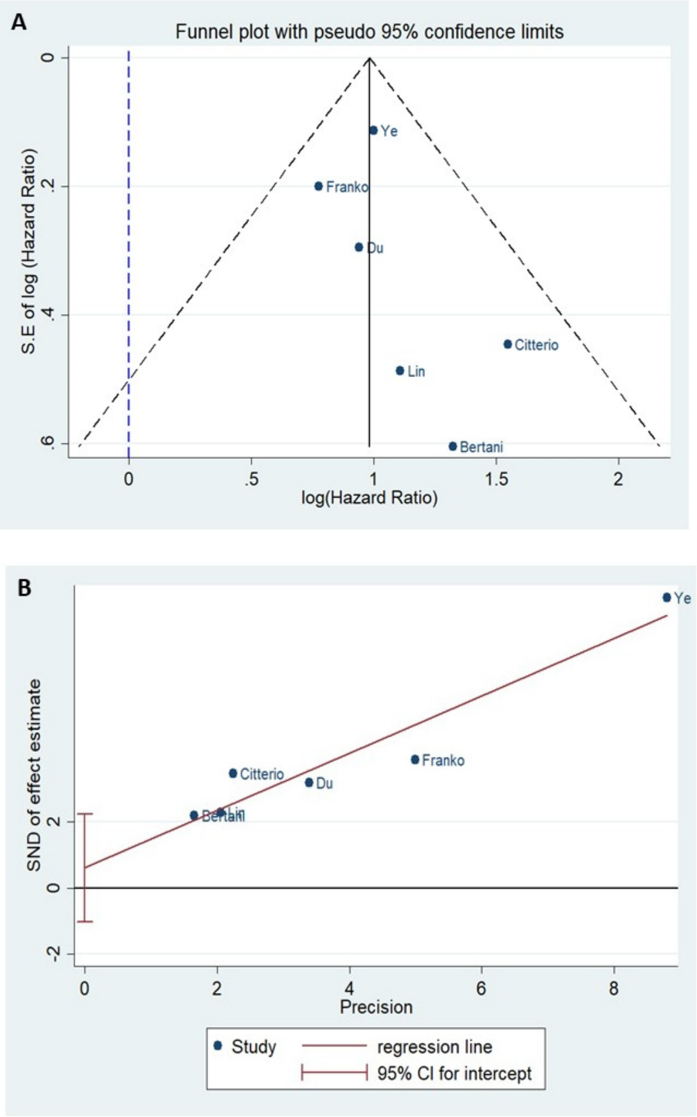

Figure S2. A: Funnel plot for studies included in the comparison of stage IV PanNEN patients undergoing non-surgical treatment vs. patients receiving palliative resection of primary tumor (PPTR) with respect to adjusted hazard ratios (HRs) overall survival analysis and **B**: Egger´s plot for studies included in this analysis.

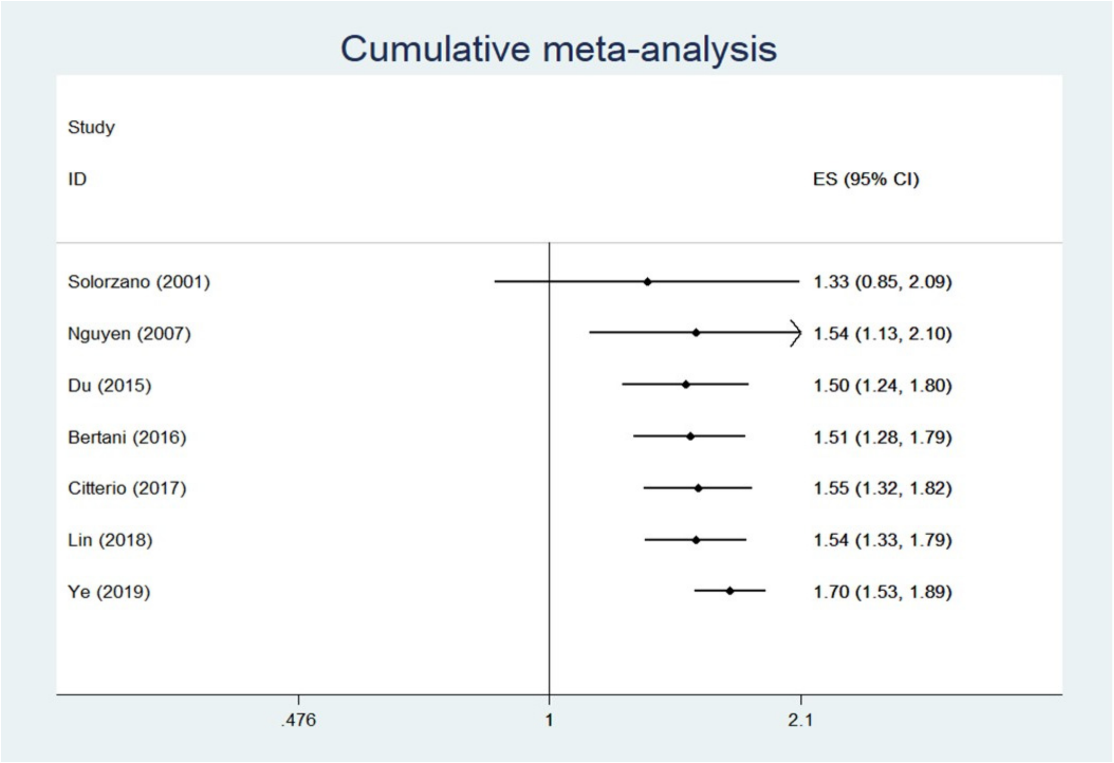

Figure S3. Cumulative unadjusted survival meta-analysis by year of publication with respect to 5-year overall survival analysis.

© 2020 by the authors. Licensee MDPI, Basel, Switzerland. This article is an open access article distributed under the terms and conditions of the Creative Commons Attribution (CC BY) license (http://creativecommons.org/licenses/by/4.0/).
